# Sphingosine Kinase-1-Dependent and -Independent Inhibitory Effects of *Zanthoxyli* Fructus to Attenuate the Activation of Mucosal Mast Cells and Ameliorate Food Allergies in Mice

**DOI:** 10.1155/2012/862743

**Published:** 2012-06-07

**Authors:** Xiaoyu Wang, Natsuko Kageyama-Yahara, Shusaku Hayashi, Takeshi Yamamoto, Makoto Kadowaki

**Affiliations:** Division of Gastrointestinal Pathophysiology, Institute of Natural Medicine, University of Toyama, 2630 Sugitani, Toyama 930-0194, Japan

## Abstract

Food allergy (FA) is relatively a common disease in infants, but effective drug therapies are not yet available. Notably, mucosal mast cells, but not connective-tissue mast cells, play important roles in food allergic reactions via the release of inflammatory mediators. Therefore, we screened medicinal herb extracts for *in vitro* and *in vivo* antiallergic activity through inhibiting mucosal mast cell activation. As a result, both antigen-induced and calcium ionophore-induced degranulation was significantly inhibited by *Zanthoxyli* Fructus water extract (ZF) in mucosal-type murine bone marrow-derived mast cells (mBMMCs). ZF suppressed the antigen-induced [Ca^2+^]_*i*_ elevation and the antigen-enhanced mRNA expression of TNF-*α*, IL-4, and IL-13. The transcriptome and real-time PCR analyses revealed that ZF greatly decreased the antigen-enhanced expression level of sphingosine kinase 1 (Sphk1), which plays a key role in the Fc*ε*RI-mediated immune responses in mast cells. Furthermore, ZF inhibited allergic symptoms in an ovalbumin-caused murine FA model and decreased the number of infiltrating mucosal mast cells and the enhanced mRNA expression levels of IL-4 and Sphk1 in the FA mice colons. These results indicate that ZF suppresses mucosal mast cell activities mainly through Sphk1-dependent mechanism, and ZF is utilized for the development of a novel, potent anti-FA agent.

## 1. Introduction

Food allergy (FA) is an abnormal immunologic reaction to food proteins. The prevalence of FA in children is estimated to be approximately 4% to 10%. The greatest prevalence is in the first few years of life, and a gradual decrease occurs during the first decade as tolerance develops [1], although some peanut allergy is rarely outgrown [2].

Although the underlying pathogenic mechanisms of food allergy are not well understood, it is well known that mast cells play central roles in the pathogenesis of various allergic disorders. The activation of mast cells leads to the secretion of various proteases, autacoids, growth factors, cytokines, and chemokines [3, 4]. Two distinct populations of mast cells exist: mucosal mast cells and connective-tissue mast cells. Considerable evidence has demonstrated that mucosal mast cells are morphologically, biochemically, and functionally distinct from connective-tissue mast cells [5]. We have previously demonstrated that the number of mucosal mast cells in the colons of our ovalbumin-induced FA model mice is increased [6], and that mucosal mast cells play important roles in the allergic reaction in our FA model [7]. At present, connective tissue mast cell stabilizers (tranilast, ketotifen, and cromolyn) are frequently used for the treatment of various allergic disorders besides FA. These agents have no effect on mucosal mast cells [8]. Until now, there has been little information on the inhibitory agents against mucosal mast cell activation [8–11].

Currently, no therapeutic drugs for FA have been developed, and the avoidance of food antigens is the central therapy that is used to prevent further reactions in FA patients. Consequently, novel approaches are being explored to develop innovative medicines. Specifically, traditional medicines have proven to be a potential source for the development of new therapeutic medicines [12]. Many herbal medicines are used as traditional remedies for treating various diseases, which include allergic diseases, particularly in Asian countries. Li et al. [12–14] have reported that Chinese herbal medicines, food allergy herbal formula-1 (FAHF-1) and FAHF-2, ameliorate peanut-induced anaphylaxis in a murine FA model. Furthermore, FAHF-2 reduces allergen-stimulated basophil activation, hyperreleasability, and percentages of circulating basophils in a 6-month clinical trial for patients with food allergy [15]. In addition, we have demonstrated that kakkonto, a traditional medicine that is frequently used in Japan and China, alleviates the allergic symptoms that are induced by food antigens [6]. Therefore, to address the development of new therapeutic medicines for FA, we screened 80 medicinal herbs that are frequently used in Japan using rat-basophilic-leukemia (RBL-) 2H3 mast-like cells. Of the 80 medicinal herbs, the water extracts of *Arecae* Semen,* Cinnamoni *Cortex,* Curcumae* Rhizoma,* Rhei* Rhizoma, and* Zanthoxyli *Fructus significantly inhibited antigen-induced degranulation. Furthermore, we found that *Zanthoxyli *Fructus water extract exhibited the most potent inhibitory effect in a preliminary study that used our FA model.

*Zanthoxylum piperitum* De Candolle (ZPDC), a deciduous shrub, is distributed in Japan, China, and Korea. The fruit of ZPDC, which is called Shan-Jiao in Chinese and Sansho in Japanese, is utilized as a spice and a traditional herbal medicine in Asia. It has been reported that glycoproteins in the *Zanthoxyli *Fructus have anti-inflammatory properties [16]. However, it remains still unclear whether *Zanthoxyli *Fructus plays a suppressive effect on the activation of mucosal mast cells and the development of FA.

In this study, we investigated the effect of the water extract of the fruit of ZPDC (ZF) on mucosal mast cells, explored the underlying mechanism of the pharmacological effects, and demonstrated for the first time that ZF inhibited the activation of mucosal mast cells *in vitro* and *in vivo* primarily through the suppression of antigen-induced sphingosine kinase 1 (Sphk1) gene expression. Therefore, ZF may serve as a leading candidate for the development of novel anti-FA agents.

## 2. Materials and Methods

### 2.1. Animals

Male BALB/c mice (5 weeks old) were purchased from Japan SLC Inc. (Shizuoka, Japan). All mice were housed with free access to food and water in the experimental animal facility at the University of Toyama. All of the animal care procedures and experiments were approved by the Animal Experiment Committee at the University of Toyama (Authorization no. S-2009 INM-9).

### 2.2. Reagents

Recombinant murine SCF, recombinant murine IL-3, recombinant murine IL-9, and TGF-*β*1 were purchased from Peprotech (London, UK). N, N-dimethylsphingosine (DMS), 3-O-caffeoylquinic acid, catechin, hyperfine, and epicatechin were purchased from Cayman Chemical (Ann Arbor, MI, USA). Limonene, A23187, ovalbumin (OVA, faction V), propidium iodide (PI), and aluminum hydroxide gel were purchased from Sigma (St. Louis, MO, USA). Cyclosporin A, W-7, and KN-93 were purchased from Calbiochem (San Diego, CA, USA). RPMI-1640 medium was purchased from Wako (Osaka, Japan). Antidinitrophenyl (DNP) IgE was purchased from Yamasa (Tokyo, Japan). Fura-2 AM and Fluo-3 AM were purchased from Dojindo (Kumamoto, Japan). Mouse GeneChip Gene 1.0 ST Array was purchased from Affymetrix (Santa Clara, CA, USA). Antiserum against mouse mucosal mast cell protease (mMCP)-1 was purchased from Moredun Scientific (Penicuik, UK). Hydroxy-*α*-sanshool was provided by Tsumura Co. (Tokyo, Japan). Sepasol Super was purchased from Nacalai Tesque (Kyoto, Japan). RNeasy Plus Micro was procured from Qiagen (Hilden, Germany).

### 2.3. Preparation of Extracts

The water extracts of medicinal herbs were provided by the Joint Usage/Research Center for Science-Based Natural Medicine, Institute of Natural Medicine, University of Toyama and the Knowledge Cluster Initiative Program (Second Stage) from the Ministry of Education, Culture, Sports, Science and Technology of Japan. Briefly, crude herbs (45.0 g) were extracted with 900 mL of hot water under reflux conditions. Following filtration through a cotton-inserted funnel, the extract was lyophilized for 2 days. The yield of ZF was 19.8% (w/w). Quality control of ZF was performed by an LC-MS analysis using a LC-IT-TOF mass spectrometer equipped with an ESI interface (Shimadzu LC-IT-TOF MS ESI, Shimadzu Scientific Instruments, Kyoto, Japan). The mass spectrometry data that were obtained from the extract were registered in the Wakan-Yaku DataBase system at the Institute of Natural Medicine of the University of Toyama (http://wakandb.u-toyama.ac.jp/wiki/LCMS:Zanthoxyli_Fructus/10025907). 

### 2.4. Cell Culture

The RBL-2H3 cells were kindly provided by Dr. Hidetaka Yakura (Tokyo Metropolitan Institute for Neuroscience, Tokyo, Japan) and maintained in complete RPMI-1640 medium. The mucosal-type murine bone marrow-derived mast cells (mBMMCs) were prepared from the femurs of BALB/c mice as described previously [9, 17]. Briefly, the bone marrow cells were cultured in complete RPMI-1640 medium that contained 40 ng/mL SCF, 20 ng/mL IL-3, 5 ng/mL IL-9, and 1 ng/mL TGF-*β*1 for 4 weeks. The mast cell purity was examined by flow cytometry (FACS Calibur; Becton Dickinson, Franklin Lakes, NJ, USA), and more than 98% of the nonadherent cells were high-affinity IgE receptor (Fc*ε*RI) and c-kit positive (data not shown).

### 2.5. Activation of RBL-2H3 Cells and mBMMCs

The activation of RBL-2H3 cells and mBMMCs was performed as previously described [9]. Briefly, the RBL-2H3 cells and mBMMCs were sensitized with 0.5 *μ*g/mL and 1.5 *μ*g/mL anti-DNP IgE for 24 h and 6 h, respectively, at 37°C. The cells were washed and incubated with medicinal herb extracts or test drugs. After 30 min, the cells were stimulated with 100 ng/mL DNP-BSA at 37°C for 1 h; the samples were then centrifuged, and the supernatants and cell pellets were collected for use in the degranulation assay, real-time PCR analysis, and transcriptome analysis. In the experiments that utilized calcium ionophore, the RBL-2H3 cells and mBMMCs were incubated with ZF or test drugs for 30 min, and the cells were then stimulated with 25 *μ*M calcium ionophore A23187 for 30 min.

### 2.6. Degranulation Assay

The degree of degranulation was assessed by measuring *β*-hexosaminidase release, which has been previously described [9]. The extent of degranulation was calculated by dividing the absorbance of the supernatant by the sum of absorbances of the supernatant and cell lysate.

### 2.7. Viability Analysis by PI Staining

The mBMMCs that were pretreated with ZF for 90 min at 37°C in a humidified 5% CO_2_ atmosphere were washed twice with fluorescence-activated cell sorter (FACS) buffer (1% BSA and 0.2% NaN_3_ in PBS), and the cells were then resuspended in FACS buffer in polystyrene round-bottom tubes. After staining with 2 *μ*g/mL PI, the viability of the cells was examined using the FACS Calibur system.

### 2.8. Intracellular Calcium Measurement

Cross-linking of Fc*ε*RI by antigens induces mast cell activation, which leads to the elevation of the intracellular calcium concentration ([Ca^2+^]*_i_*) prior to the exocytosis of granules and cytokines [4]. The measurement of [Ca^2+^]*_i_* was performed as previously described [8]. Briefly, the sensitized mast cells were loaded with 5 *μ*M Fura-2 AM in loading buffer. The fluorescence was measured at 340 and 380 nm using a model F-4500 fluorescence spectrophotometer intracellular Ca^2+^ measurement system (Hitachi, Tokyo, Japan), and the background-corrected 340 : 380 ratio was calibrated.

For an experiment with calcium ionophore A23187, the calcium imaging experiments were performed using the AQUACOSMOS IMAGO CCD camera-based system (HAMAMATSU, C7773, Hamamatsu, Japan). The cells were loaded with 10 *μ*M Fluo-3 AM in loading buffer for 30 min; they were washed once and were then monitored for 4 min at 310 nm.

### 2.9. Transcriptome Analyses

The global gene expression analysis was performed using an Affymetrix Mouse GeneChip Gene 1.0 ST Array that was spotted with 28,853 probe sets. The total RNA was isolated from mBMMCs using the RNeasy Plus Micro kit according to the manufacturer's instructions, and the mRNA for the array hybridization was prepared as described in the GeneChip Expression Technical Manual. The data were first analyzed using the GeneChip Analysis Suite Software (Affymetrix) and were further analyzed using Gene-Spring software (Silicon Genetics, CA, USA) to identify the significant genes. The fold-change log_2_ ratio is the change in the expression level of a transcript that is expressed as the log_2_ ratio (the fold-change log_2_ ratio of 1 and −1 are equal to a fold change of 2 and 0.5, resp.).

### 2.10. Allergic Diarrhea Induction in a Murine FA Model

Male BALB/c mice were sensitized twice at a 2-week interval by intraperitoneal injection with 50 *μ*g of OVA in the presence of 1.3 mg of aluminum hydroxide gel as an adjuvant. Two weeks after systemic priming, the mice were repeatedly given 50 mg of OVA that was dissolved in 0.3 mL of water using intragastric feeding needles every other day. Diarrhea was assessed by visually monitoring the mice for up to 1 h following the intragastric challenge. For treatment with ZF (32, 100 and 320 mg/kg body weight) or DMS (1 mg/kg body weight) in the FA model, the agents were orally administered to the mice each day throughout the period of oral OVA administration, and they were administered 1 h prior to the OVA challenge.

For histological analysis, the proximal colon was excised 1 h after the fifth or sixth oral OVA challenge and stained using antibodies against mMCP-1 according to the procedure that was described previously [6].

### 2.11. Real-Time PCR

Total RNA was extracted from the proximal colon using the Sepasol Super according to the manufacturer's instructions. Quantitative real-time PCR was performed as previously described [6]. The following primer pairs were used: IL-4 forward: 5′-GGT CTC AAC CCC CAG CTA GT-3′ and reverse: 5′-GCC GAT GAT CTC TCT CAA GTG AT-3′; IL-13 forward: 5′-GGA TAT TGC ATG GCC TCT GTA AC-3′ and reverse: 5′-AAC AGT TGC TTT GTG TAG CTG A-3′; TNF-*α* forward: 5′-AAG CCT GTA GCC CAC GTC GTA-3′ and reverse: 5′-GGC ACC ACT AGT TGG TTG TCT TTG-3′; Sphk1 forward: 5′-AGT CGC CAG ACA CCC TCC TG-3′ and reverse: 5′-CCT CGA GGG CAT TCT GGT TCC-3′; GAPDH forward: 5′-TGA CCA CAG TCC ATG CCA TC-3′ and reverse: 5′-GAC GGA CAC ATT GGG GGT AG-3′. The target mRNA was normalized to GAPDH mRNA as an internal control in each sample. Results were expressed as the relative ratio to the control group average (1 in vehicle-treated mBMMCs without DNP-BSA stimulation or the proximal colons of vehicle-treated FA mice).

### 2.12. Statistical Analysis

Data are presented as the mean ± SD. The statistical comparisons were performed using Student's *t*-test and repeated measures one-way ANOVA followed by post hoc Dunnett's test or chi-square test with SPSS software (version 19, IBM, Somers, NY, USA). Values of *P* < 0.05 were considered significant.

## 3. Results

### 3.1. Water Extracts of *Arecae* Semen, *Cinnamoni* Cortex, *Curcumae* Rhizoma, *Rhei* Rhizoma, and *Zanthoxyli* Fructus Inhibit Antigen-Stimulated Degranulation in RBL-2H3 Cells

**Figure 1:**
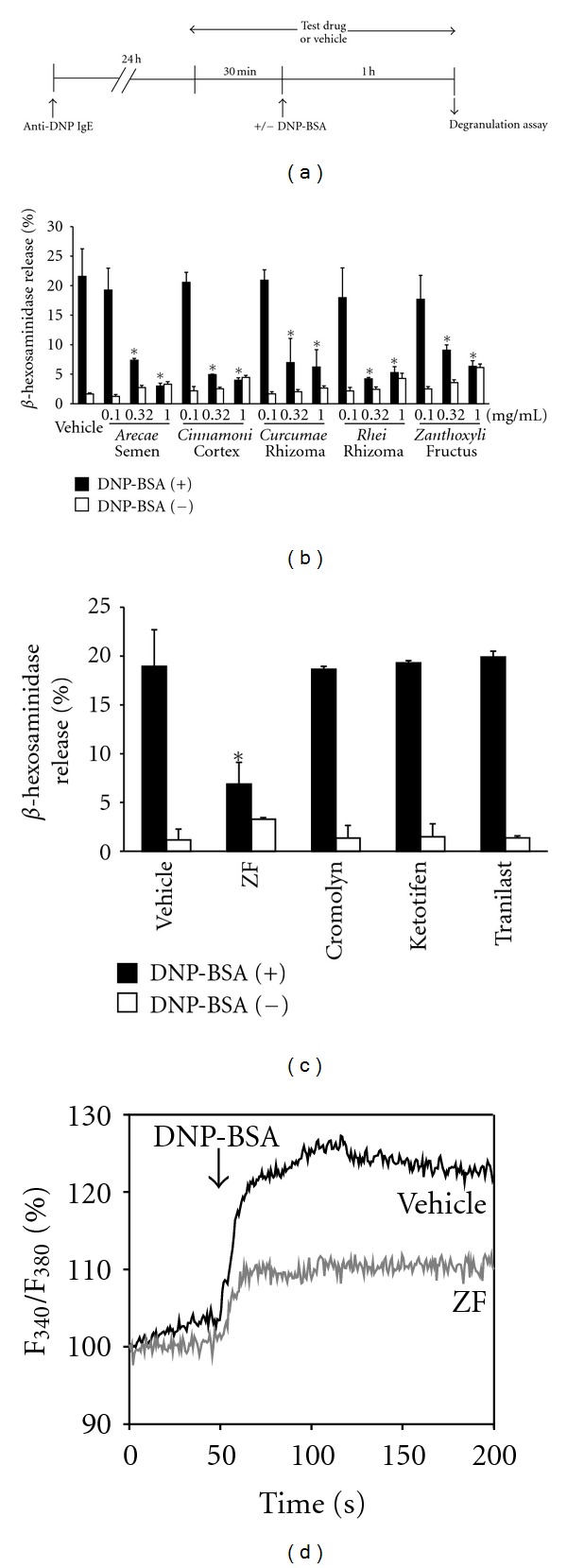
Inhibitory effect of the water extracts of *Arecae* Semen,* Cinnamoni *Cortex,* Curcumae* Rhizoma,* Rhei* Rhizoma, and* Zanthoxyli *Fructus on degranulation that was induced by antigens in the RBL-2H3 cells. (a) The time scheme for the degranulation assay. (b) RBL-2H3 cells that were sensitized with 0.5 *μ*g/mL anti-DNP IgE (24 h) were incubated with herbal medicine extracts for 30 min. The cells were stimulated with (filled) or without (open) 100 ng/mL DNP-BSA for 1 h, and *β*-hexosaminidase release was determined. (c) The inhibitory effect of antiallergic drugs (10 *μ*M) on DNP-BSA-induced degranulation was examined. The data are expressed as the mean ± SD (*n* = 3; B, C). *P < 0.05 compared with the vehicle (b, c). (d) The effect of ZF (0.32 mg/mL) treatment on the increase of [Ca^2+^]*_i_* that was induced DNP-BSA. The RBL-2H3 cells that were sensitized with IgE were labeled with Fura-2 AM and treated with ZF or vehicle for 30 min. The Ca^2+^-mobilization was determined after stimulation with DNP-BSA using F4500. The data are representative of at least three independent experiments.

Eighty medicinal herb extracts were screened for their inhibitory effect on DNP-BSA-stimulated degranulation in RBL-2H3 cells. The water extracts of *Arecae* Semen, *Cinnamoni* Cortex,* Curcumae* Rhizoma,* Rhei* Rhizoma, and* Zanthoxyli *Fructus (≥0.1 mg/mL) inhibited the stimulated degranulation in a dose-dependent manner (Figure 1(b)). In contrast, none of the clinically available antiallergic drugs (10 *μ*M), cromolyn, ketotifen and tranilast, which are known to be connective tissue mast cell stabilizers, inhibited the degranulation (Figure 1(c)).

In an* in vivo* preliminary study, ZF (500 mg/kg body weight) displayed the most potent inhibitory effect of the five extracts that were examined in our FA model (data not shown). Therefore, we selected ZF for further study.

The mobilization of intracellular calcium is essential for degranulation and cytokine production in mast cells. As shown in Figure 1(d), ZF attenuated the DNP-BSA-induced [Ca^2+^]*_i_* elevation in RBL-2H3 cells. In addition, ZF did not alter Fc*ε*RI surface expression in RBL-2H3 cells (the expression was 96.3% of that of nontreated RBL-2H3), which indicates that ZF inhibits signals downstream of Fc*ε*RI cross-linking.

Additionally, we examined the inhibitory effects of ZF constituents hydroxy-*α*-sanshool, limonene, 3-O-caffeoylquinic acid, catechin, hyperine, and epicatechin (1, 10, and 100 *μ*M). Individually, hydroxyl-*α*-sanshool, epicatechin, catechin, and hyperine slightly attenuated the degranulation only at the highest concentration (100 *μ*M) (Table 1).

### 3.2. ZF Inhibits Antigen-Induced Activities in mBMMCs

**Figure 2:**
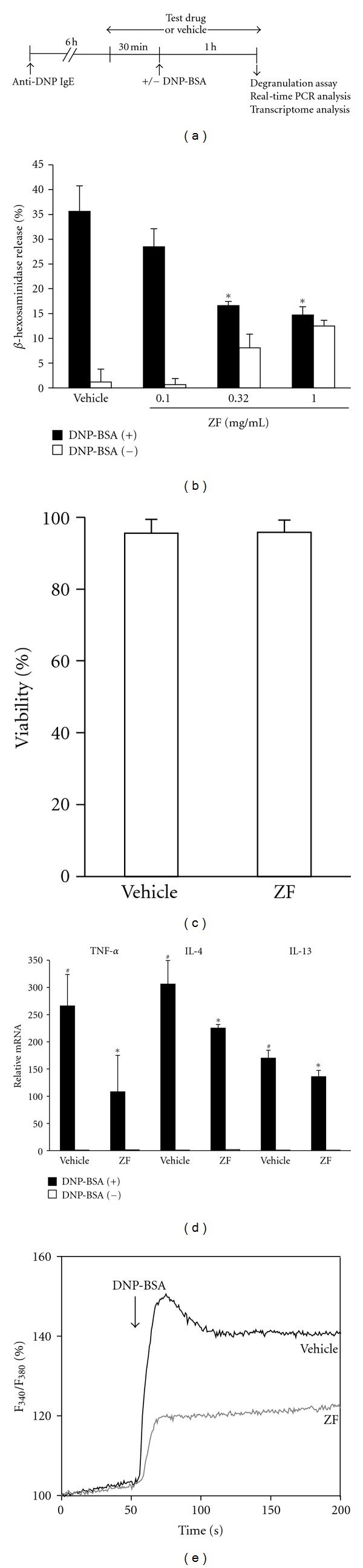
The effect of ZF (0.32 mg/mL) on degranulation, cytokine levels, and [Ca^2+^]*_i_* elevation of the mBMMCs that were stimulated with DNP-BSA. (a) The time scheme for the degranulation assay, real-time PCR analysis, and transcriptome analysis. (b) The mBMMCs that were sensitized with 1.5 *μ*g/mL anti-DNP IgE (6 h) were incubated with ZF for 30 min. The cells were stimulated with (filled) or without (open) DNP-BSA (1 h), and the *β*-hexosaminidase release was determined. (c) The mBMMCs were pretreated with ZF (0.32 mg/mL) for 90 min and stained with PI; the viability was analyzed using the FACS Calibur system. (d) The sensitized mBMMCs were incubated with ZF (0.32 mg/mL) or vehicle for 30 min and then stimulated with DNP-BSA for 1 h, and the total RNA was extracted. The mRNA levels of TNF-*α*, IL-4, and IL-13 were analyzed by real-time PCR. The results are expressed as the relative ratio to the vehicle-treated mBMMCs without DNP-BSA stimulation. The data are expressed as the mean ± SD (*n* = 3–4; b, c, d). ^#^ *P* < 0.05 compared with DNP-BSA (−) (d), *P < 0.05 compared with the vehicle (b, d). (e) The sensitized mBMMCs were labeled with Fura-2 AM for 30 min and incubated with ZF or vehicle for 30 min. Ca^2+^-mobilization was determined following stimulation with DNP-BSA. The data are representative of at least three independent experiments.

In a dose-dependent manner, ZF significantly inhibited the degranulation of the mBMMCs that were induced with DNP-BSA (Figure 2(b)) without detectably affecting the viability of the mBMMCs (Figure 2(c)). As shown in Figure 2(d), mRNA expression of TNF-*α*, IL-4, and IL-13 was extremely enhanced by DNP-BSA. ZF (0.32 mg/mL) significantly suppressed the increased expression of TNF-*α*, IL-4, and IL-13. In contrast, the expression of these genes was not affected by ZF in the mBMMCs that were not stimulated with DNP-BSA (unactivated mBMMCs). In addition, ZF attenuated the DNP-BSA-induced [Ca^2+^]*_i_* increases (Figure 2(e)).

### 3.3. ZF Reduces Sphk1 mRNA Expression in Antigen-Stimulated mBMMCs

To investigate the pharmacological mechanism that is responsible for the inhibitory effect of ZF on mBMMCs, we examined the global mRNA expression profiles of normal mBMMCs, DNP-BSA-stimulated mBMMCs (activated mBMMCs), and ZF-pretreated activated mBMMCs (ZF mBMMCs) using an Affymetrix Mouse GeneChip Array. The expression levels of 49 genes were elevated >2-fold higher in the activated mBMMCs when compared to the normal mBMMCs, and they were decreased by >50% in the ZF mBMMCs when compared to the activated mBMMCs. In contrast, the expression levels of 6 genes were decreased by >50% in the activated mBMMCs when compared to the normal mBMMCs, and they were elevated >2-fold higher in the ZF mBMMCs when compared to the activated mBMMCs (Table 2). Interestingly, the expression of Sphk1, which plays an important role in antigen-induced degranulation [18–20], was induced approximately 5.7-fold in the activated mBMMCs when compared with the normal mBMMCs. The ZF pretreatment greatly reduced the expression of Sphk1 to 16% of the expression level in the activated mBMMCs, whereas the DNP-BSA stimulation did not alter Sphk2 expression when compared with that in the normal mBMMCs (1.35-fold relative to the normal mBMMCs).

**Figure 3:**
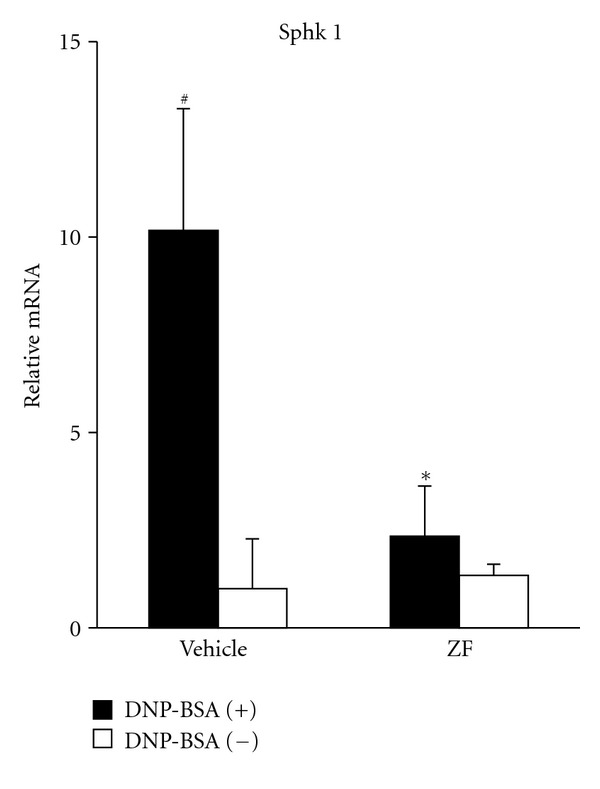
The effect of ZF (0.32 mg/mL) on the mRNA expression levels of Sphk1. The sensitized mBMMCs were incubated with ZF or vehicle for 30 min and were stimulated with (filled) or without (open) DNP-BSA. The mRNA levels of Sphk1 were analyzed by real-time PCR. The results are expressed as the relative ratio to the vehicle-treated mBMMCs without DNP-BSA stimulation. The data are expressed as the mean ± SD (*n* = 3). ^#^ *P* < 0.05 compared with DNP-BSA (−), *P < 0.05 compared with the vehicle.

Furthermore, we quantitatively assessed the suppressive effect of ZF on Sphk1 mRNA expression using real-time PCR. As shown in Figure 3, the Sphk1 mRNA expression level in the activated mBMMCs was markedly enhanced 10.2-fold relative to the unactivated mBMMCs. The induction was dramatically reduced to approximately 20% in the ZF mBMMCs. In contrast, the Sphk1 mRNA expression was not affected by ZF in the unactivated mBMMCs.

**Figure 4:**
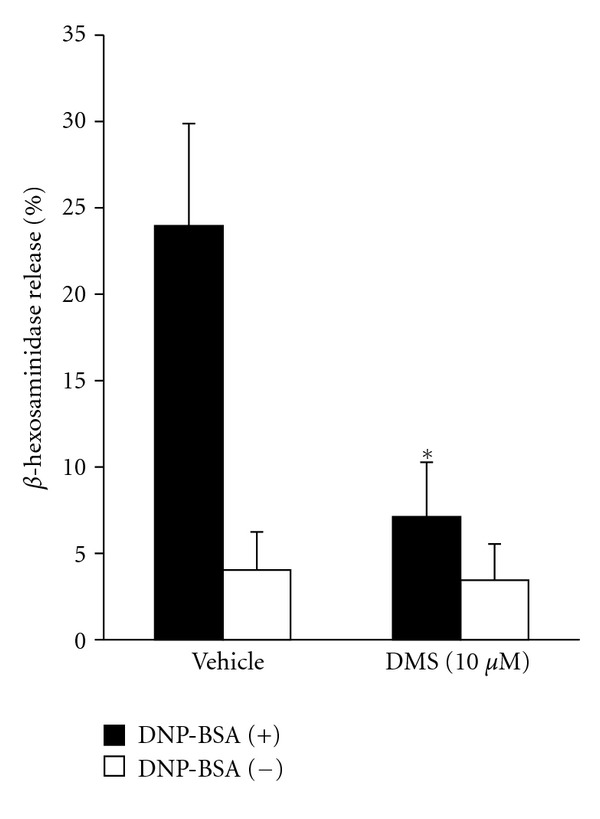
The inhibitory effect of DMS on the degranulation of mBMMCs that were induced by DNP-BSA. The sensitized mBMMCs were pretreated with 10 *μ*M DMS for 30 min prior to DNP-BSA stimulation. The data are expressed as the mean ± SD (*n* = 4). *P < 0.05 compared with the vehicle.

To clarify the involvement of Sphk1 in the degranulation of mucosal mast cells, we examined the effect of DMS, which is a specific Sphk inhibitor, on the antigen-induced degranulation in mBMMCs. DMS (10 *μ*M) significantly suppressed the release of *β*-hexosaminidase (Figure 4).

### 3.4. ZF Inhibits Calcium Ionophore-Induced Degranulation

**Figure 5:**
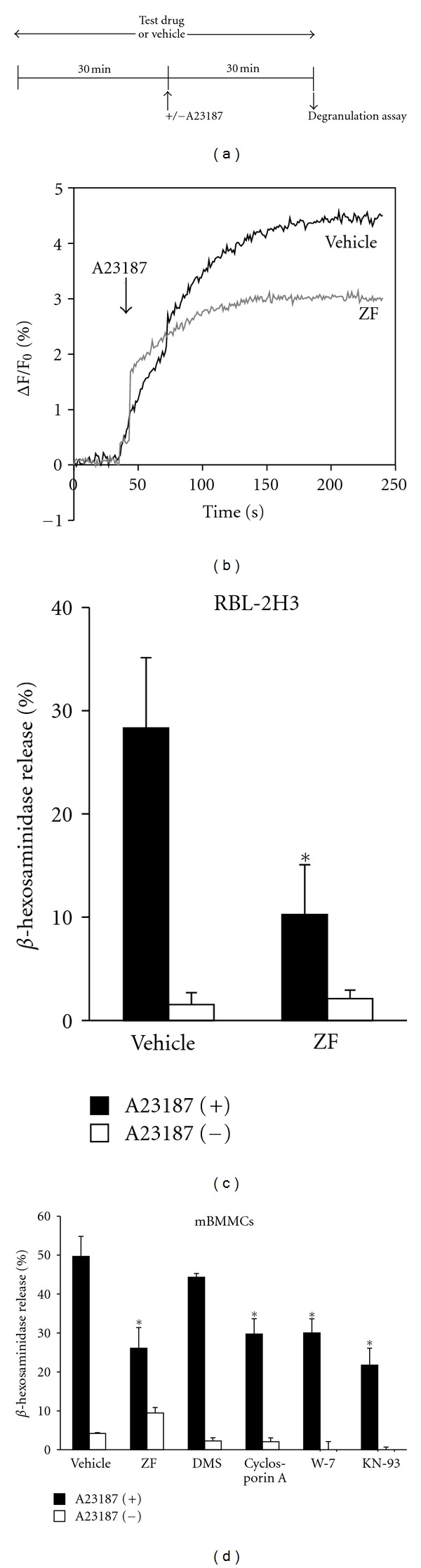
The effect of ZF (0.32 mg/mL) treatment on A23187-induced [Ca^2+^]*_i_* elevation and degranulation. (a) The time scheme for the degranulation assay. (b) RBL-2H3 cells were labeled with Fluo-3 AM and incubated with ZF or vehicle for 30 min. The cells were stimulated with 25 *μ*M A23187 and monitored by calcium imaging. The data are representative of at least three independent experiments. (c) The RBL-2H3 cells were incubated with ZF or vehicle for 30 min, the cells were stimulated with (filled) or without (open) A23187 for 30 min, and *β*-hexosaminidase release was determined. (d) The mBMMCs were incubated with ZF (0.32 mg/mL), DMS (10 *μ*M), cyclosporin A (1 *μ*M), W-7 (32 *μ*M), KN-93 (32 *μ*M), or vehicle for 30 min, and the cells were stimulated with 25 *μ*M A23187 for 30 min. *β*-hexosaminidase release was determined. The data are expressed as the mean ± SD (*n* = 4; c, d). *P < 0.05 compared with the vehicle (c, d).

To investigate the pharmacological profile of ZF on mucosal mast cell activation, we examined the effect of ZF on the [Ca^2+^]*_i_* increases and *β*-hexosaminidase release induced by the calcium ionophore A23187. The ZF treatment diminished the [Ca^2+^]*_i_* increases in RBL-2H3 cells (Figure 5(b)). In addition, ZF significantly inhibited A23187-induced degranulation in both RBL-2H3 cells and mBMMCs (Figures 5(c), 5(d)). Moreover, the calcineurin inhibitor cyclosporin A (1 *μ*M), the calmodulin antagonist W-7 (32 *μ*M), and the Ca^2+^/calmodulin-dependent protein kinase II inhibitor KN-93 (32 *μ*M) inhibited A23187-induced degranulation in mBMMCs to a similar extent as ZF, which indicates that A23187 induces degranulation via both calcineurin- and calmodulin-dependent pathways in mBMMCs (Figure 5(d)). In contrast, DMS (10 *μ*M) did not affect A23187-induced degranulation in mBMMCs (Figure 5(d)). Furthermore, when the mBMMCs were stimulated with the calcium ionophore A23187 (instead of anti-DNP IgE and DNP-BSA) and examined with the Affymetrix Mouse Genechip Array, the expression of Sphk1 was not altered (data not shown).

### 3.5. ZF Suppresses Allergic Symptoms in the Murine FA Model

**Figure 6:**
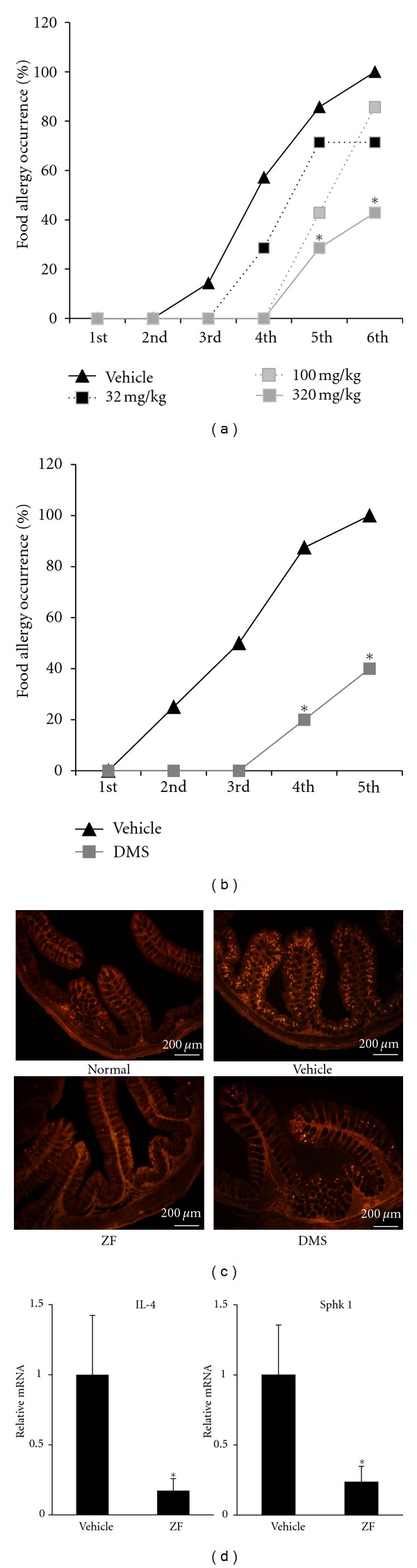
The effect of ZF and DMS on the development of FA *in vivo*. (a) The induction of allergic diarrhea was compared between the vehicle- and ZF-treated groups. ZF was orally administered during the induction of allergic diarrhea by OVA oral challenge as described in the Materials and Methods section (*n* = 8). (b) DMS (1 mg/kg) suppressed the incidence of allergic diarrhea (*n* = 5–8). (c) The proximal colons of the vehicle, ZF (320 mg/kg), and DMS (1 mg/kg) treated mice after oral challenge with OVA were stained with anti-mMCP1 antibody. The scale bar represents 200 *μ*m. (d) The expression levels of IL-4 and sphk1 in the proximal colons were measured using real-time PCR. The results are expressed as the relative ratio to the proximal colons of the vehicle-treated FA mice. The data are expressed as the mean ± SD (*n* = 5). *P < 0.05 compared with the vehicle.

In OVA-challenged mice (FA mice), allergic diarrhea began to occur after the third oral OVA challenge (Figure 6(a)). The administration of ZF (320 mg/kg) significantly reduced the incidence of OVA-induced diarrhea, and the incidence of diarrhea was decreased to approximately 40% after the sixth OVA challenge. As shown in Figure 6(b), the administration of DMS (1 mg/kg) likewise significantly reduced the incidence of diarrhea. Furthermore, the number of mucosal mast cells that were observed by immunohistochemistry with mMCP-1 antibody dramatically increased in the proximal colons of FA mice compared with the number in normal mice, which is consistent with our previous paper [6]. ZF and DMS dramatically decreased the number of infiltrating mucosal mast cells in the proximal colons of the FA mice (Figure 6(c)). In addition, as shown in Figure 6(d), ZF repressed the mRNA expression of IL-4 and Sphk1 in the proximal colons of the FA mice.

## 4. Discussion

In this study, we have demonstrated *in vitro* and *in vivo* that ZF inhibited Fc*ε*RI cross-linking-induced mucosal mast cell activation; this inhibition was primarily achieved via the suppression of Sphk1 mRNA expression, and Sphk1 plays a key role in Fc*ε*RI-mediated immune responses in mucosal mast cells. Furthermore, ZF suppressed the calcium ionophore-induced degranulation, which could not be suppressed by an Sphk1 inhibitor. Therefore, the present results suggest that ZF could be utilized for the development of a novel, potent anti-FA agent.

### 4.1. Inhibitory Effect of ZF on Antigen-Induced Mucosal Mast Cell Activation *In Vitro*

Mast cells are key players in various allergic responses, and mucosal mast cells have been shown to play pivotal roles in gastrointestinal hypersensitivity [6–8]. They respond to both IgE-dependent (antigen) and IgE-independent (such as bacterial toxins and neurotransmitters) stimulation and release a wide variety of bioactive mediators into adjacent tissues [21].

Although the available mast cell stabilizers did not affect degranulation in RBL-2H3 cells as well as mBMMCs, ZF inhibited antigen-induced degranulation in mBMMCs and RBL-2H3 cells. Mast cells generate and release proinflammatory and Th2-related cytokines, including TNF-*α*, IL-4, and IL-13, in response to various stimuli [3]. TNF-*α* is largely responsible for allergic inflammation, and IL-4, and IL-13 are necessary for the development of Th2 immune responses and the induction of IgE class switching [4]. ZF significantly inhibited the mRNA expression of these cytokines in the mBMMCs. Lee et al. have recently reported in a connective-tissue mast cell model that glycoproteins in the *Zanthoxyli* Fructus have an inhibitory effect on the release of histamine and *β*-hexosaminidase in the compound 48/80-treated human mast cells [16]. In mucosal-type mast cells, ZF displayed potent inhibitory effects on degranulation and proinflammatory- and Th2-related cytokine production, and ZF suppressed the increase of [Ca^2+^]*_i_* that was triggered by antigens in RBL-2H3 cells and mBMMCs. These results suggest that ZF inactivates mucosal mast cells by suppressing [Ca^2+^]*_i_*.

Individually, the constituents of ZF that were tested here did not significantly inhibit antigen-induced degranulation in RBL-2H3 cells. One possible explanation for this result is that the inhibitory effect of ZF on mast cells is related to the additive/synergistic effects of these and other constituents in ZF. However, further studies on the active constituents of ZF will be required to fully explain the inhibitory effect of ZF.

### 4.2. Suppression of Sphk1 mRNA Expression by ZF and the Pathophysiological Role of Sphk1 in Mucosal Mast Cells *In Vitro*

To investigate the mechanism underlying the inhibitory effect of ZF on mucosal mast cell activation, we performed global transcriptional profiling of mBMMCs by DNA microarray. We found that the expression level of Sphk1 mRNA was increased in the antigen-stimulated mBMMCs and decreased following ZF treatment. The stimulation of mast cells by antigens induces two mammalian Sphks (Sphk1 and Sphk2) to generate sphingosine 1-phosphate (S1P). S1P has been demonstrated to function intracellularly as a second messenger for the regulation of cell survival, cell proliferation, and intracellular Ca^2+^ mobilization [22, 23]. A recent study shows that Sphk1 but not Sphk2 plays a critical role in the antigen-induced [Ca^2+^]*_i_* elevation, degranulation, and cytokine production in connective-tissue type BMMCs [18]. Furthermore, antigen-induced degranulation is suppressed by the deletion of Sphk1 in RBL-2H3 cells and human mast cells [19, 20]. In the present study, our results revealed that the Sphk inhibitor DMS significantly suppressed antigen-induced degranulation in mBMMCs. Thus, Sphk1 is a determinant of the responsiveness to the cross-linking of Fc*ε*RI with antigen in mucosal mast cells as well as connective-tissue mast cells. Furthermore, ZF decreased the antigen-induced elevation of [Ca^2+^]*_i_* through the suppression of antigen-induced enhancement of Sphk1 mRNA expression *in vitro*. Therefore, Sphk1 may be a new therapeutic target for diseases that are caused by both types of mast cells; ZF may provide an opportunity to develop a novel therapeutic strategy for the treatment of FA. To date, Sphk inhibitor DMS and the S1P receptor modulator FYT720 have been developed, while there is no information about drugs that suppress the expression of Sphk1. Taken together, these data suggest that ZF may provide a prototype for therapeutic drugs for the treatment of various allergic diseases, particularly FA.

### 4.3. Sphk1-Independent Mechanism Underlying the Inhibitory Effect of ZF on Mucosal Mast Cells *In Vitro*

We found that the calcium ionophore A23187 did not alter Sphk1 mRNA expression in mBMMCs using a transcriptome analysis (our unpublished data), and DMS did not inhibit A23187-induced degranulation in mBMMCs, which is consistent with previous reports. Both the DMS treatment and Sphk1 knock-down fail to affect ionomycin-induced degranulation in RBL-2H3 cells or connective-tissue-type BMMCs [18, 19]. Taken together, these data suggest that Sphk1 acts at the upstream of the calcium signaling pathway in mast cells. In contrast, ZF suppressed A23187-induced degranulation and [Ca^2+^]*_i_* elevation in mucosal-type mast cells. Furthermore, in mBMMCs, the calcineurin inhibitor cyclosporin A, the calmodulin antagonist W-7, and the Ca^2+^/calmodulin-dependent protein kinase II inhibitor KN-93 suppressed A23187-induced degranulation to a similar extent as ZF, which is consistent with previous reports [24, 25]. These results indicate that A23187 induces degranulation via both calcineurin- and calmodulin-dependent pathways in mast cells. Taken together, these data suggest that ZF suppresses the activation of mast cells by blocking calcium influx with or without inhibiting calcineurin- and calmodulin-dependent pathways in mast cells. Therefore, our results indicate that ZF suppresses not only antigen-induced Sphk1 expression, but also critical molecules in the calcium influx pathway. Additional studies will be required to understand this inhibitory mechanism.

### 4.4. Therapeutic Effect of ZF and Inhibition of the Sphk1 Pathway in the Murine FA Model *In Vivo*

The oral administration of ZF suppressed the occurrence of allergic diarrhea and decreased the number of colonic mucosal mast cells in our FA model. We showed that the administration of ZF downregulated the mRNA expression of IL-4 in the proximal colons of FA mice, which suggests that ZF suppressed the Th2-polarized cytokine profile in the proximal colons of FA mice. In addition, ZF inhibited the expression of Sphk1 mRNA in the proximal colons of FA mice. Furthermore, in the present study, DMS likewise reduced the occurrence of allergic diarrhea and the number of mucosal mast cells in the proximal colons of FA mice. Similarly, DMS exerts an inhibitory effect on the OVA-induced pulmonary inflammatory responses in a murine model of allergic asthma [26]. It is generally accepted that the activation and degranulation of mast cells that are induced by antigens and the migration of mast cells toward antigens are important pathogenic mechanisms in allergic diseases. In the present study, we demonstrated that the number of mucosal mast cells was greatly increased in the proximal colons of FA mice, and the increase was reduced following the treatment with ZF or DMS. However, the mechanism by which these mucosal mast cells migrate to sites of allergy reaction in the colon is not fully understood. It has been reported that DMS can inhibit the movement of RBL-2H3 cells toward antigens [19]. Furthermore, the downregulation of Sphk1 expression using small interfering RNA completely blocks the migration toward antigens in RBL-2H3 cells [19] and human mast cells [20], whereas the downregulation of Sphk2 has no effect [19, 20]. Thus, these findings suggest that ZF downregulates Sphk1 expression and thereby suppresses both the activation of mast cells that are induced by the antigens and the migration of mast cells toward the antigens. Furthermore, *Zanthoxyli *Fructus is one of the herbal constituents in FAHF-1 and FAHF-2 which show inhibition of systemic anaphylaxis and suppression of histamine release in a murine model of food allergy [13, 14]. In particular, FAHF-2 has inhibitory effects on peripheral blood basophils from patients with food allergy [15]. These previous studies may further support the findings of this study that ZF may have a potential for food allergy treatment. Taken all together, it is suggested that ZF may provide a novel strategy for the treatment of FA via inhibition of the Sphk pathway.

 Although the active compounds in ZF have not yet been identified, ZF provides novel insights into the development of a therapeutic drug against both Sphk1-dependent and -independent mechanisms of FA that are closely related to the activity of mucosal mast cells. Furthermore, ZF or its active constituents may provide a novel prototype for antiallergic agents that act via the inhibition of Sphk1-dependent and -independent pathways.

## Figures and Tables

**Table 1:** The inhibitory activity of ZF components (100 *μ*M) on antigen-induced degranulation. The data are expressed as the mean ± SD (*n* = 3).

Compounds	Inhibition of degranulation (%)
Hydroxy-*α*-sanshool	21.1 ± 11.9
Epicatechin	15.7 ± 7.1
Catechin	13.0 ± 4.8
Hyperoside	7.7 ± 5.2
Limonene	−11.4 ± 9.9
3-O-Caffeoylqunic acid	−13.3 ± 7.7

**Table 2:** List of genes selected by microarray analysis. The fold-change log_2_ ratio was calculated as the log_2_ ratio of the expression level in the activated mBMMCs compared to the expression in the normal mBMMCs or as the log_2_ ratio of the expression level in the ZF mBMMCs compared to that in the activated mBMMCs. (a) Upregulation (>2 fold) of genes by DNP-BSA stimulation and downregulation (>2 fold) after ZF pretreatment. (b) Downregulation (>2 fold) of genes by DNP-BSA stimulation and upregulation (>2 fold) after ZF pretreatment.

Gene name	Gene description	Fold-change log_2_ ratio	
		IgE + DNP/Normal	ZF/IgE + DNP
(a)			
Tnip3	TNFAIP3 interacting protein 3	4.47	−3.36
Sphk1	Sphingosine kinase 1	2.50	−2.65
Chac1	ChaC, cation transport regulator-like 1 (*E. coli*)	1.80	−2.40
Trib1	Tribbles homolog 1 (*Drosophila*)	3.21	−2.39
Uhrf1bp1l	UHRF1 (ICBP90) binding protein 1-like	1.82	−2.23
Car2	Carbonic anhydrase 2	2.16	−1.86
Socs3	Suppressor of cytokine signaling 3	1.89	−1.82
Sema7a	Sema domain, immunoglobulin domain (Ig), and GPI membrane anchor, (semaphorin) 7A	2.25	−1.77
Fermt2	Fermitin family homolog 2 (*Drosophila*)	2.81	−1.75
Zfp57	Zinc finger protein 57	1.90	−1.71
Klf9∣Gm9971	Kruppel-like factor 9 ∣ predicted gene 9971	1.42	−1.68
Mustn1	Musculoskeletal, embryonic nuclear protein 1	1.57	−1.64
Tnfsf8	Tumor necrosis factor (ligand) superfamily, member 8	2.48	−1.64
Gimap5	GTPase, IMAP family member 5	1.41	−1.61
Dusp18	Dual-specificity phosphatase 18	1.08	−1.58
Slco4a1	Solute carrier organic anion transporter family, member 4a1	2.25	−1.57
Fhl2	Four and a half LIM domains 2	1.06	−1.56
Spry2	Sprouty homolog 2 (*Drosophila*)	2.64	−1.53
A630033H20Rik	RIKEN cDNA A630033H20 gene	2.34	−1.52
Zc3h12a	Zinc finger CCCH type containing 12A	1.70	−1.51
Nfkbia	Nuclear factor of kappa light polypeptide gene enhancer in B-cells inhibitor, alpha	1.92	−1.50
Il2	Interleukin 2	1.91	−1.50
Zc3h12c	Zinc finger CCCH type containing 12C	2.28	−1.47
Marcksl1	MARCKS-like 1	2.88	−1.42
Hbegf	Heparin-binding EGF-like growth factor	4.90	−1.41
Csf2	Colony stimulating factor 2 (granulocyte macrophage)	5.30	−1.41
Erf	Ets2 repressor factor	2.10	−1.40
Kcnk5	Potassium channel, subfamily K, member 5	1.13	−1.38
Mmd	Monocyte to macrophage differentiation associated	2.05	−1.37
Phlda1	Pleckstrin homology-like domain, family A, member 1	3.10	−1.32
Zc3h12c	Zinc finger CCCH type containing 12C	2.26	−1.31
Rilpl2	Rab interacting lysosomal protein-like 2	1.48	−1.31
Faah	Fatty acid amide hydrolase	2.35	−1.29
Fst∣Thrap3	Follistatin ∣ thyroid-hormone-receptor-associated protein 3	1.67	−1.19
Insig1	Insulin-induced gene 1	1.65	−1.19
Spry1	Sprouty homolog 1 (*Drosophila*)	4.36	−1.19
Pde12	Phosphodiesterase 12	1.29	−1.19
Rc3h1	RING CCCH (C3H) domains 1	1.13	−1.18
Dusp2	Dual-specificity phosphatase 2	2.15	−1.15
F3	Coagulation factor III	1.16	−1.13
Eda2r	Ectodysplasin A2 receptor	1.65	−1.11
Prrg4	Proline-rich Gla (G-carboxyglutamic acid) 4 (transmembrane)	1.95	−1.11
Traf6	TNF receptor-associated factor 6	1.03	−1.08
Peli1	Pellino 1	1.48	−1.07
Cxcl2	Chemokine (C-X-C motif) ligand 2	3.91	−1.04
Ptger4	Prostaglandin E receptor 4 (subtype EP4)	1.06	−1.03
Siah2	Seven in absentia 2	1.20	−1.02
Chka	Choline kinase alpha	1.58	−1.01
Ehd4	EH-domain containing 4	1.10	−1.01

(b)			
Cbfa2t3	Core-binding factor, runt domain, alpha subunit 2, translocated to 3 (human)	−1.59	1.71
ATP6∣Gm10925	ATP synthase F0 subunit 6 ∣ predicted gene 10925	−1.20	2.01
Nynrin	NYN domain and retroviral integrase containing	−1.11	1.00
Hist1h1c	Histone cluster 1, H1c	−1.01	1.15
Arrdc3	Arrestin domain containing 3	−1.10	1.36
Gfi1b	Growth-factor-independent 1B	−1.52	1.40
